# Supplementary material for: Determinants of overweight and obesity among children between 5 to 11 years in Ecuador: A secondary analysis from the National Health Survey 2018
Source: PLoS One. 2024 Apr 5;19(4):e0296538. doi: 10.1371/journal.pone.0296538 (PMC10997090; doi:10.1371/journal.pone.0296538)
Supplement: S2 Table — Each category’s percentages highlight the proportional differences between the two groups. (DOCX) [file pone.0296538.s003.docx]

**S2 Table. Characteristics comparison between non-overweight/non-obese and overweight/obese children. Each category's percentages highlight the proportional differences between the two groups.**

|  | **Non-overweight or obese children**  **n=6876** | | **Overweight or obese children^b^**  **n=3931** | |
| --- | --- | --- | --- | --- |
| **Variable** | **No.** | **Weighted % or mean (SE)** | **No.** | **Weighted % or mean (SE)** |
| Sex (Male) | 3390 | 48.3 | 2151 | 53.7 |
| Age of the children | 6876 | 7.9 (0.04) | 3931 | 8.3 (0.05)^c^ |
| Ethnicity |  |  |  |  |
| Ethnicity (Indigenous) | 894 | 7.6 | 358 | 6.6 |
| Ethnicity (Afroecuadorian) | 296 | 4.7 | 163 | 4.8 |
| Ethnicity (Mestizo) | 5325 | 80.3 | 3211 | 81.7 |
| Ethnicity (White) | 81 | 1.4 | 60 | 1.0 |
| Ethnicity (Montubio or other) | 280 | 6.0 | 139 | 5.9 |
| Economic quintiles by income^a^ |  |  |  |  |
| Economic quintile by income (1st quintile) | 2016 | 28.2 | 849 | 20.6 |
| Economic quintile by income (2nd quintile) | 1524 | 24.6 | 820 | 21.9 |
| Economic quintile by income (3rd quintile) | 1297 | 18.9 | 799 | 23.0 |
| Economic quintile by income (4th quintile) | 1041 | 15.3 | 726 | 19.1 |
| Economic quintile by income (5th quintile) | 920 | 13.0 | 688 | 15.5 |
| Education of the children (Elementary school ongoing) | 6824 | 99.5 | 3918 | 99.8 |
| Regular class attendance (Yes) | 6741 | 98.1 | 3879 | 98.5 |
| The head of the household receives the BDH (Yes) | 321 | 4.8 | 134 | 3.9 |
| Number of people in the household | 6876 | 5.1 (0.04) | 3931 | 4.8 (0.05)^d^ |
| Inadequate disposal of excreta (Yes) | 1762 | 26.2 | 808 | 20.5 |
| Regular physical activity (Yes) | 948 | 14.6 | 441 | 11.5 |
| Perception of low consumption of vegetables (Yes) | 3366 | 47.9 | 1852 | 45.6 |
| Days per week of consumption in fast food restaurants | 6876 | 0.9 (0.03) | 3931 | 1.0 (0.03)^d^ |
| Days per week of school food consumption | 6876 | 4.4 (0.04) | 3931 | 4.4 (0.04)^d^ |
| The child buys food at school (Yes) | 4519 | 63.2 | 2752 | 65.7 |
| The child eats the food provided by the school (Yes) | 4613 | 66.1 | 2490 | 61.2 |
| Family members recognize, understand, and use the labeling of processed foods (Yes) | 4098 | 65.1 | 2537 | 66.0 |
| In the family, they reduced the consumption of processed foods with a red label | 2629 | 47.2 | 1627 | 50.4 |
| BDH=Human Development Voucher, by its Spanish spelling.  SE= Standard error.  **^a^** Income quintiles are calculated at the household level using monetary labour income per capita, first calculating the total income for each income earner. This total income includes earnings from work, income from investments, transfers, and other benefits, such as cash social transfers. Once we add all these up, we obtain the total household income. Then, we determine the average income per person (per capita income) by dividing the total household income by the number of people in each household. Subsequently, the population is systematically arranged on the basis of the per capita income variable. The calculation of the quintiles was performed by dividing the population into five equal groups, known as quintiles. The first quintile includes the percentage of households with the lowest income, the second quintile includes the next percentage, and so on until the fifth quintile, which includes the percentage of households with the highest income.  ^b^ Overweight and obesity were determined by calculating the Body Mass Index (BMI), adjusted for age and sex, according to the WHO growth references. Overweight is BMI-for-age greater than 1 standard deviation above the WHO Growth Reference median; and obesity is greater than 2 standard deviations above the WHO Growth Reference median.  ^c^ T test  ^d^ U Mann Whitney test. The rest of the statistical tests were Chi^2^. | | | | |
