# Supplementary material for: Determinants of overweight and obesity among children between 5 to 11 years in Ecuador: A secondary analysis from the National Health Survey 2018
Source: PLoS One. 2024 Apr 5;19(4):e0296538. doi: 10.1371/journal.pone.0296538 (PMC10997090; doi:10.1371/journal.pone.0296538)
Supplement: S3 Table — (DOCX) [file pone.0296538.s004.docx]

**S3 Table. Adjusted Odds Ratios of overweight or obesity from each explanatory variable using the parsimonious logistic regression model of Table 2 after excluding: (i) people categorized in the lowest income quintile, (ii) people categorized in the highest income quintile, (iii) people who receive the BDH, (iv) people within the upper third of the highest number of people per household.**

| **Variable** | **Excluding children categorized in the highest income quintile**  **n= 9199** | **p-value** | **Excluding children whose parents received the BDH**  **n= 10352** | **p-value** | **Excluding children within the upper third of the highest number of people per household**  **n= 8273** | **p-value** |
| --- | --- | --- | --- | --- | --- | --- |
| Male sex (female is the ref.) | 1.27 (1.23 to 1.31) | <0.001 | 1.28 (1.25 to 1.31) | <0.001 | 1.27 (1.13 to 1.47) | <0.001 |
| Age of the child (per each increase in one year) | 1.0 (1.08 to 1.10) | <0.001 | 1.10 (1.09 to 1.10) | <0.001 | 1.10 (1.09 to 1.12) | <0.001 |
| Ethnicity |  |  |  |  |  |  |
| Ethnicity (Indigenous is the ref.) | 1 | - | 1 | - | 1 | - |
| Ethnicity (Afroecuadorian) | 1.13 (0.98 to 1.30) | 0.082 | 1.11 (1.00 to 1.23) | 0.044 | 1.12 (1.00 to 1.25) | 0.048 |
| Ethnicity (Mestizo) | 1.14 (1.01 to 1.29) | 0.037 | 1.10 (1.04 to 1.17) | 0.002 | 1.08 (0.95 to 1.23) | 0.232 |
| Ethnicity (White) | 1.46 (1.08 to 1.97) | 0.014 | 1.26 (1.00 to 1.58) | 0.046 | 1.33 (1.14 to 1.56) | <0.001 |
| Ethnicity (Montubio or other) | 0.95 (0.76 to 1.19) | 0.669 | 0.88 (0.77 to 1.02) | 0.086 | 0.94 (0.81 to 1.08) | 0.386 |
| Economic quintiles by income^a^ |  |  |  |  |  |  |
| Economic quintile by income (1st quintile is the ref.) | 1 | - | 1 | - | 1 | - |
| Economic quintile by income (2nd quintile) | 1.16 (1.12 to 1.21) | <0.001 | 1.16 (1.14 to 1.19) | <0.001 | 1.21 (1.15 to 1.27) | <0.001 |
| Economic quintile by income (3rd quintile) | 1.32 (1.10 to 1.59) | 0.003 | 1.31 (1.10 to 1.57) | 0.002 | 1.38 (1.14 to 1.67) | 0.001 |
| Economic quintile by income (4th quintile) | 1.39 (1.16 to 1.65) | <0.001 | 1.37 (1.17 to 1.62) | <0.001 | 1.44 (1.10 to 1.88) | 0.008 |
| Economic quintile by income (5th quintile) | - | - | 1.38 (1.29 to 1.49) | <0.001 | 1.39 (1.28 to 1.52) | <0.001 |
| p-for-trend | 1.12 (1.04 to 1.20) | 0.002 | 1.09 (1.05 to 1.13) | <0.001 | 1.09 (1.04 to 1.15) | 0.001 |
| Number of people in the household (per each extra person) | 0.93 (0.91 to 0.95) | <0.001 | 0.93 (0.92 to 0.95) | <0.001 | 0.89 (0.88 to 0.91) | <0.001 |
| Inadequate disposal of excreta (otherwise is the ref.) | 0.85 (0.77 to 0.95) | 0.003 | 0.82 (0.76 to 0.89) | <0.001 | 0.89 (0.81 to 0.96) | 0.005 |
| Regular physical activity (otherwise is the ref.) | 0.78 (0.77 to 0.79) | <0.001 | 0.78 (0.73 to 0.88) | <0.001 | 0.73 (0.68 to 0.80) | <0.001 |
| Consumption of food provided by the school (otherwise is the ref.) | 0.93 (0.80 to 1.07) | 0.284 | 0.93 (0.82 to 1.05) | 0.232 | 0.92 (0.80 to 1.05) | 0.195 |
| Family members recognize, understand, and use the labeling of processed foods (otherwise is the ref.) | 1.16 (1.0 to 1.36) | 0.055 | 1.16 (1.03 to 1.31) | 0.015 | 1.13 (1.00 to 1.29) | 0.058 |
| BDH=Human Development Voucher, by its Spanish spelling  ^a^ Income quintiles are calculated at the household level using monetary labour income per capita, first calculating the total income for each income earner. This total income includes earnings from work, income from investments, transfers, and other benefits, such as cash social transfers. Once we add all these up, we obtain the total household income. Then, we determine the average income per person (per capita income) by dividing the total household income by the number of people in each household. Subsequently, the population is systematically arranged on the basis of the per capita income variable. The calculation of the quintiles was performed by dividing the population into five equal groups, known as quintiles. The first quintile includes the percentage of households with the lowest income, the second quintile includes the next percentage, and so on until the fifth quintile, which includes the percentage of households with the highest income. | | | | | | |
